# Solid fuels use for cooking and sleep health in adults aged 45 years and older in China

**DOI:** 10.1038/s41598-021-92452-0

**Published:** 2021-06-25

**Authors:** Haiqing Yu, Jiajun Luo, Kai Chen, Krystal J. Godri Pollitt, Zeyan Liew

**Affiliations:** 1 Department of Environmental Health Sciences, Yale School of Public Health, 60 College Street, New Haven, CT 06510 USA; 2 Yale Center for Perinatal, Pediatric, and Environmental Epidemiology, Yale School of Public Health, New Haven, CT 06510 USA

**Keywords:** Environmental sciences, Environmental impact, Epidemiology

## Abstract

Outdoor air pollution has been linked to poor sleep health, but limited studies have investigated the relationship between solid cooking fuels and sleep health in adults. Therefore, we analyzed data from the China Health and Retirement Survey (CHARLS), a national survey of about 17,000 residents aged over 45. Participants were restricted to those who participated in CHARLS 2011, 2013 and 2015 (n = 8,668). Sleep health was indicated by self-reported average sleep hours at night and the numbers of unrested days/week in CHARLS 2015. We analyzed cooking fuel types reported and assessed the duration of solid fuels usage as consistent (indicated use in all three surveys or 6 + years) or inconsistent use (indicated use in one or two surveys or 1–4 years). We found consistent use of solid fuels was associated with a shorter sleep duration (OR = 1.17 95% CI 1.01, 1.35 for ≤ 6 h vs. 7–9 h/day) and higher frequencies of feeling unrested (OR = 1.32 95% CI 1.12, 1.55 for ≥ 5 days/week vs. none) compared with cleaner fuels use. The associations for inconsistent solid fuels use and sleep health were in the similar direction but smaller in magnitude. Further research is needed to confirm our findings and evaluate the exposure impact of specific fuel types to inform intervention strategies.

## Introduction

Sleep disorders have been a major public health issue, especially in an aging society, since the elderly are susceptible to age-related changes in circadian rhythm and sleep cycle^1^. Early awakening fragmented sleep, insufficient sleep, and poor sleep efficiency are common among middle-aged adults and the elderly^2,3^. Epidemiological studies have shown that sleep disorders and poor sleep quality are associated with a broad range of adverse health outcomes, including cardiovascular disease, hypertension, stroke, cognitive impairment, and depression^4–8^. Potential mechanisms suggested explaining these associations include that poor sleep health impaired carbohydrate metabolism and endocrine function, activated inflammatory processes, and affected the sympathetic nervous system’s activity to increase blood pressure^9–11^. An effective intervention on sleep problems may prevent and mitigate the development of other severe chronic diseases.

A variety of physical and psychosocial risk factors have been identified for sleep disorders, including lifestyles such as lack of exercise and smoking^12,13^, and psychological factors such as job stress and depression^14,15^. In addition to these individual factors, environmental factors can also affect sleep health. Artificial light at night and noise pollutions have been suggested to disturb sleep^16–19^. Moreover, accumulating evidence has also suggested that exposures to ambient air pollution, including particulate matter mass concentration (PM_10_, PM_2.5_), sulfur dioxide (SO_2_), nitrogen dioxide (NO_2_) and carbon monoxide (CO), could affect sleep health^20,21^ by inducing systemic inflammations in the respiratory tract or central nervous system^22,23^. Epidemiological evidence has predominantly derived from studies assessing outdoor air pollutions^24–27^. For example, a most recent study of 395,651 elderly Chinese in Ningbo Province found short-term exposures to outdoor air pollutants, including NO_2_, SO_2_, ozone (O_3_), PM_2.5_ and PM_10_ mass concentration were associated with hospital visits for sleep disorders^25^. Limited studies have investigated the association between indoor air quality and sleep health^28^. A study evaluating bedroom environment among 63 middle-aged adults in Thailand reported an elevation in 1-year mean PM_10_ concentration was associated with an increase in apnea–hypopnea and respiratory disturbance indexes^29^. Two small-scale intervention studies in Peru had reported that the improvement of biomass fuel stoves could improve sleep quality among children^30,31^. Finally, two recent studies from specific regions of China suggested household air pollution from solid fuel combustion affected poor sleep quality in elderly aged 80 and above^32^ and that emissions from cooking oil combustion affected sleep quality in middle-aged adults^33^.

Solid fuels use for cooking activities can be a major source of indoor air pollutants, including PM, NO_2_, SO_2_ and CO. At present, about 3 billion people, mostly from low- or middle-income countries, are using solid fuels and kerosene for cooking because of poor financial situation or lack of infrastructure^34^. According to a survey of 512,891 adults from 10 areas across China during 2004–2008, about half of the participants (52%) reported using coals or charcoal for cooking or heating, especially for rural participants^35^. In addition, the aging population is rapidly growing in China^36^. Older adults are likely to spend more time indoor^37^, and they are vulnerable to ambient pollutions and susceptible to chronic health issues because of a decline in overall physical health^38,39^. Within this context, we conducted a study to evaluate the association between household use of solid fuels for cooking and sleep health using a large and representative sample of Chinese residents above the age of 45 years.

## Materials and methods

### Data source

We analyzed data from the China Health and Retirement Longitudinal Study (CHARLS, http://charls.pku.edu.cn/zh-CN), a nationally representative longitudinal survey of about 17,000 Chinese residents ages 45 and older^40^. The study was launched by Peking University in 2011 which has collected data in 150 counties/districts and 450 villages/resident committees across China every two year. Participants were guided to fill out the self-administered questionnaires by trained researchers. The ethics approval for the CHARLS was granted by the Ethics Review Committee of Peking University and all methods were carried out in accordance with relevant guidelines and regulations. All the participants provided signed informed consent at the time of participation.

Our analysis focused on CHARLS data from 2011 to 2015. Only participants who completed all three waves of surveys (CHARLS 2011, 2013, and 2015) were included in the main analyses to evaluate the duration of exposure prior to outcome assessment (N = 9484). After excluding participants with brain damage or mental retardation and with emotional, nervous, or psychiatric problems that could compromise self-reported data (N = 816), the final sample size for analysis was 8668.

### Solid fuels use for cooking

In all three CHARLS surveys from 2011 to 2015, participants were asked: What is their main source of cooking fuel? We classified coal and crop residue/wood burning as solid fuels, and liquefied petroleum gas (LPG), natural gas, marsh gas, and electricity as cleaner fuel types^41,42^. According to the responses from all three surveys, we further classified solid fuels usage as consistent (indicated use in all three surveys or 6 + years) or inconsistent use (indicated use in one or two surveys only or 1–4 years) prior to sleep outcome assessment in 2015^41^.

### Sleep health

In all three CHARLS surveys, participants were asked (1) During the past month, how many hours of actual sleep did you get at night (average hours for one night)? (2) How many days were in accord with the state of feeling unrested from my sleep?^42^ Sleep health data from 2015 survey was used in the primary analyses to study consistency of solid fuels exposure influences on sleep. Based on the responses, we classified the average hours of sleep into severe insufficient sleep (< 6 h/night), mild insufficient sleep (6–7 h/night), sufficient sleep (7–9 h/night as the reference) and excessive sleep (> 9 h/night), while the number of days feeling unrested were categorized as none or rarely (< 1 day / week), sometimes (1–2 days/week ), moderately (3–4 days/week), and most or all of the time (5–7 days/week)^41,42^.

### Statistical analysis

We conducted multinomial logistic regression to estimate the odds ratio (OR) and 95% confidence interval (CI) for sleep duration or days feeling unrested according to solid fuels usage in cooking, adjusting for potential confounding factors. For sleep duration, sufficient sleep (7–9 h/night) was set as the reference group and for days feeling unrested those reported none or rarely or none (< 1 day) was used as the reference. Our main analyses evaluated the cumulative impacts of exposure on sleep measures. We compared sleep outcomes among consistent (6 + years) or inconsistent solid fuels usage (1–4 years) during the study period with never solid fuels use (or only cleaner fuels use) as the reference. We calculated *p* value for trend using a continuous exposure variable based on the cumulative years of solid fuels use during the survey periods (value 0, 2, 4, and 6 years assuming each survey wave covered usage in the past two years). In addition, we assessed the cross-sectional associations between solid fuels use and sleep outcomes reported using data from each study wave from 2011 to 2015 separately.

Potential confounders were selected based on literature reviews considering factors associated with choices of cooking fuels use and sleep health and data availability in CHARLS. In all analyses, we adjusted for sex (male or female), age (45–65 or > 65 years), urbanicity of the living place (urban or rural), household economic level estimated by expenditure ($\le$ 10,000, 10,000–50,000, 50,000–100,000, > 100,000, RMB), education level (less than high school, high school, college degree or higher), marital status (married and living with a spouse; yes or no), active or passive smoking (yes or no) , and cooking location within household (whether the kitchen is located outside the living room; yes or no). Urban or rural area was classified based on the codes of National Bureau of Statistics of China. The rural–urban fringes and villages were classified into urban areas which include residential households that use solid fuels. Household economic level was measured by overall household expenditure in 2014, including clothing and bedding, traveling expenses, centrally heated fees, consumption of durable goods and electronics, education, medical and fitness expenditures, taxes and donations. Household expenditure has been showed to be a reasonable measure for household economic levels in previous research^43,44^. Passive smoke exposure was measured by whether other family members in the household had smoking habits. Less than 6.5% participants had at least one missing value of the main potential confounders and they were excluded in the statistical analyses.

In addition, we performed stratified analyses by age (45–65 or > 65 years), sex (male or female), and urbanicity of the residence (urban or rural) when estimating the effect of consistent solid fuels use on sleep health. Sleep problems are more common among older adults and women are more likely to responsible for cooking in Chinese families^45^. The patterns of exposure and sleep problems might also differ in urban or rural China^35^. Tests of heterogeneity in multiplicative scale were also performed by assessing the *p* value of the interaction term for the exposure variable and the potential effect modifier in the regression model.

Moreover, a previous study in CHARLS has suggested that solid fuels use was associated with depression in this population^46^. We examined the co-occurrence of sleep problems and depression in analyses by classifying participants as who experienced no sleep problems or depression, who only had either sleep problems or depression, and who had both sleep problems and depression using the 2015 survey. Participants were considered to have sleep problems when they experienced insufficient or excessive sleep ($\le 6$ h or $\ge 9$ h) and high frequency of unrested ($\ge 3$ days in a week). Depression symptoms were measured by the self-reported Center for Epidemiologic Studies Depression (CES-D) scale. According to the recommended cut-off^46,47^, participants were classified as having depression when the CES-D score was 12 or greater.

We re-evaluated our main findings by excluding participants reported use of natural gas in the cleaner fuels reference group concerning NO_x_ emissions from burning natural gas indoors^48^ and we evaluated the potential effects in each wave. To assess whether selection bias affected our main results, we compared the study characteristics among those who participated in the baseline survey (2011) with those who participated in all three study waves (2011–2015). We performed an inversed probability selection weighting (IPSW) analysis to account for the probability of participation in all three surveys predicted by baseline factors collected in 2011, including the cooking fuel types, sleep health and all covariate data included in the study^49^.

Finally, we performed a quasi-experimental analysis to estimate the exposure effect on sleep health by studying the change from solid to clean fuel types for cooking during the study period. Among self-reported solid fuel users identified in 2011 (N = 4275), we compared sleep health among those who switched to cleaner fuels assessed in later years (n = 1604) with those who used solid fuels consistently from 2011 to 2015 as the reference (n = 2671). In addition, we also compared the same reference group to those who used solid fuels in 2011 and 2013, and most recently switched to cleaner fuels use reported in 2015 (n = 743).

## Results

### Summary of selected study characteristics

**Figure 1:**
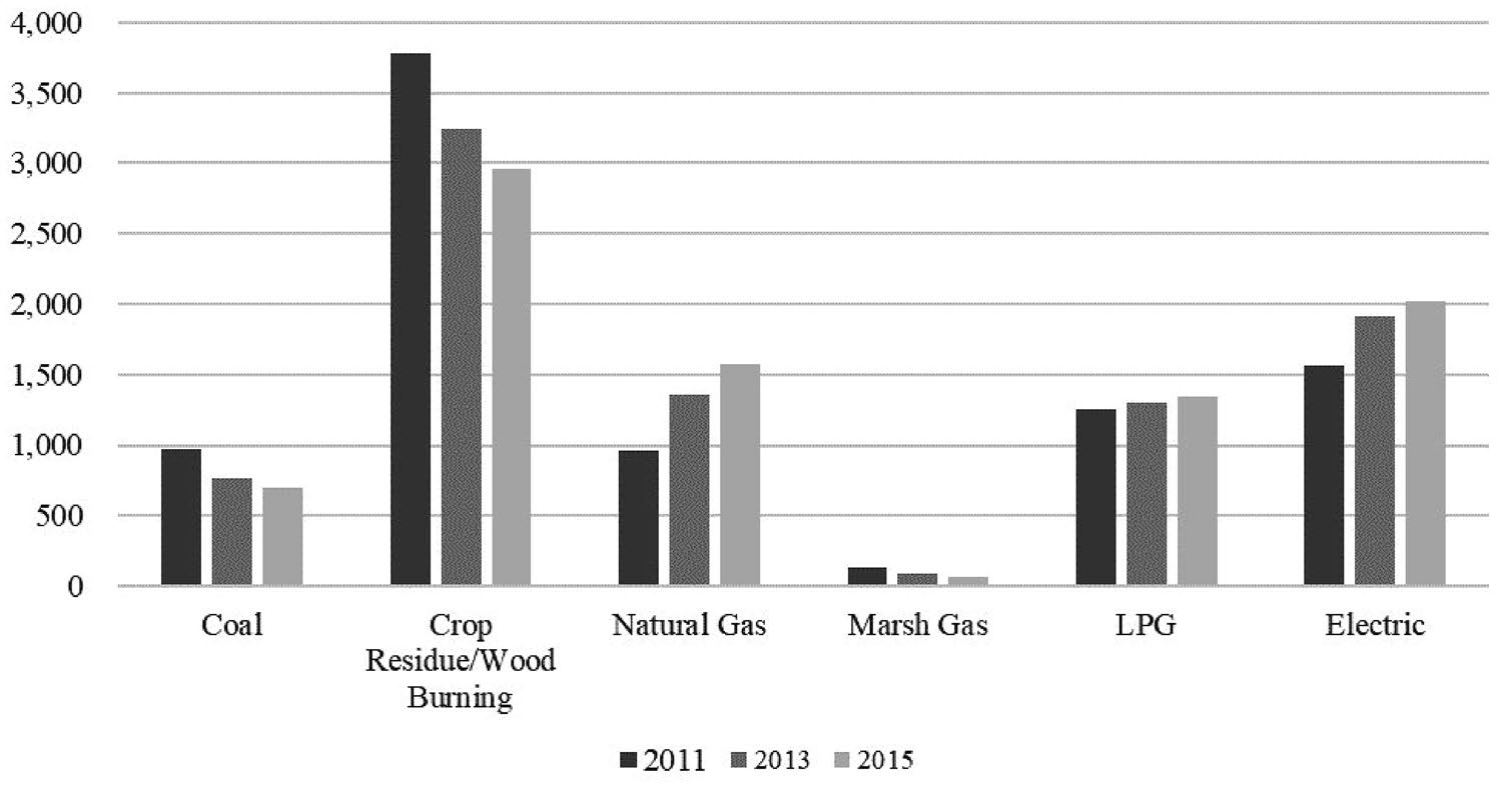
The numbers of participants on each fuel type for cooking reported in the three study waves. Coal and crop residue/wood burning were considered as solid fuels in this study. Clean fuels included natural gas, marsh gas, liquefied petroleum gas (LPG) and electric.

In this population, 64.6% (N = 5604) reported ever using solid fuels while 35.4% (N = 3064) indicated only using cleaner fuels for cooking during the study period. Among the solid fuels users, about half (47.6%) were considered consistent use throughout the study period. The distribution for the specific fuel types reported for cooking is presented in Fig. 1. The usage of solid fuel types, including coal and crop residue or wood burning for cooking has decreased from 2011 to 2015. In comparison, cleaner fuel types use including natural gas and electric have increased.

The selected characteristics of the study population by solid fuels use groups are presented in Table 1. Participants reporting consistent solid fuels use during the study period were older, had lower education level and household expenditure. As expected, participants living in rural areas were more likely to use solid fuels. More than 90% of participants had a kitchen outside the indoor living area, but those using solid fuels for a longer time were more likely not.

**Table 1:** Characteristics of study participants in the CHARLS, 2011–2015.

Characteristics	Cleaner fuels use only (N = 3064), N (%)	Solid fuels use reported in one or two surveys only (N = 2933), N (%)	Consistent solid fuels use reported in all three surveys (N = 2671), N (%)
**Age (years)**			
45–65	2265 (74.3)	2010 (68.9)	1664 (62.5)
Over 65	783 (25.7)	907 (31.1)	1000 (37.5)
Missing	16	16	7
**Sex**			
Male	1322 (48.4)	1270 (48.6)	1165 (49.1)
Female	1420 (51.6)	1346 (51.4)	1209 (50.9)
Missing	10	7	11
**Urbanicity of the living place**			
Rural	1003 (36.4)	1873 (71.5)	1993 (83.8)
Urban	1755 (63.6)	746 (28.5)	385 (16.2)
**Expenditure in 2015 (RMB)**			
≤ 10,000	1845 (61.0)	2230 (77.1)	2115 (80.2)
10,000–50,000	932 (30.8)	549 (19.0)	457 (17.3)
50,000–100,000	128 (4.2)	69 (2.4)	42 (1.6)
$>$ 100,000	118 (3.9)	44 (1.5)	24 (0.9)
Missing	41	41	33
**Educational level**			
No formal	754 (27.4)	1178 (45.0)	1282 (53.9)
≤ 12 years	1872 (67.9)	1425 (54.5)	1083 (45.5)
≥ 12 years	130 (4.7)	16 (0.6)	13 (0.6)
Missing	3	2	0
**Marital status**			
Married	2353 (85.3)	2129 (81.3)	1997 (84.0)
Not married	405 (14.7)	490 (18.7)	381 (16.0)
**Smoking**			
Active	1229 (41.3)	1264 (44.3)	1192 (45.9)
Passive	803 (27.0)	760 (26.6)	695 (26.8)
Never	944 (31.7)	829 (29.1)	711 (27.4)
Missing	88	80	73
**Cooking location within household**			
Yes	2933 (96.4)	2713 (93.2)	2424 (91.8)
No	111 (3.7)	198 (6.8)	218 (8.3)
Missing	20	22	29
**Average hours of sleep at night**			
$<$ 6	892 (29.1)	924 (31.5)	921 (34.5)
6–7	720 (23.5)	619 (21.1)	482 (18.1)
7–9	1227 (40.1)	1134 (38.7)	980 (36.7)
$>$ 9	225 (7.3)	256 (8.7)	288 (10.8)
**The number of unrested days per week**			
< 1	1522 (55.2)	1345 (51.4)	1180 (49.6)
1–2	378 (13.7)	375 (14.3)	337 (14.2)
3–4	389 (14.1)	364 (13.9)	355 (14.9)
5–7	469 (17.0)	535 (20.4)	506 (21.3)

### Associations between solid fuels use and sleep health

Consistent solid fuels use was associated with higher odds for insufficient (OR = 1.17 95% CI 1.01, 1.35) or excessive sleep (OR = 1.22 95% CI 0.97, 1.53) at night compared with participants who cooked with cleaner fuels only, while the associations for sleep duration were null for inconsistent solid fuels use compared with cleaner fuels use.

Consistent solid fuels use reported in three surveys was also associated with higher frequencies of days feeling unrested. The odd ratios were 1.26 (95% CI 1.06, 1.51) for 3–4 days/week and 1.32 (95% CI 1.12, 1.55) for more than 5 unrested days/week among consistent solid fuels users. The associations were in the similar direction but smaller in magnitude for participants indicated solid fuels use in one or two surveys only. The *p* values for cumulative years of solid fuels use and unrested sleep were all smaller than 0.10 (Table 2).

**Table 2:** Associations between solid fuels use for cooking and self-reported sleep health in the CHARLS.

Outcomes	Inconsistent solid fuels use (reported in 1 or 2 surveys) vs. cleaner fuels use	Consistent solid fuels use (reported in all surveys) vs. cleaner fuels use	*P* value for cumulative years of exposure^b^
	OR (95% CI)^a^	OR (95% CI)^a^	
**Average hours of sleep at night**			
$<$ 6	1.04 (0.91, 1.19)	1.17 (1.01,1.35)	0.11
6–7	0.96 (0.83, 1.12)	0.91 (0.77 1.07)	0.20
7–9	Reference	Reference	Reference
$>$ 9	1.03 (0.83, 1.28)	1.22 (0.97, 1.53)	0.12
**The number of unrested days per week**			
$<$ 1	Reference	Reference	Reference
1–2	1.12 (0.95, 1.33)	1.17 (0.98, 1.40)	0.06
3–4	1.06 (0.90, 1.26)	1.26 (1.06, 1.51)	0.01
5–7	1.25 (1.07, 1.46)	1.32 (1.12, 1.55)	0.01

^a^Adjusted for age, sex, education, marital status, household expenditure, active or passive smoking, urbanicity, and cooking location within household.

^b^*P* value was calculated using the cumulative years of solid fuels use exposure (0, 2, 4, and 6 years) fitted as a continuous variable.

### Associations between consistent solid fuels use and sleep health stratified by age, sex and urbanicity

In general, the associations between consistent solid fuels use and impaired sleep health appeared to be stronger for elderly 65 years and older (Table 3). Long-term solid fuels users had 55% greater odds (OR = 1.55, 95% CI 1.04, 2.30) for excessive sleep compared with cleaner fuels users among the elderly, while the effect sizes were closer to null for adults 45–65 years. For unrested days of sleep, the association between consistent solid fuels use and the most severe group of 5–7 unrested days also appeared to be stronger among the elderly (OR = 1.72 95% CI 1.26, 2.33). However, the interaction *p* values were greater than 0.10 suggested an insufficient statistical precision to conclude an effect modification by age.

**Table 3:** Associations between solid fuels use for cooking and slef-reported sleep helath, stratified by age, sex and urbancity.

Outcomes	Consistent solid fuels use (reported in all surveys) vs. cleaner fuels use only								
	Age 45–65	> 65 years	Interaction p value^b^	Men	Women	Interaction p value^b^	Rural area	Urban area	Interaction p value^b^
	OR (95% CI)^a^	OR (95% CI)^a^		OR (95% CI)^a^	OR (95% CI)^a^		OR (95% CI)^a^	OR (95% CI)^a^	
**Average hours of sleep at night**									
$<$ 6	1.14 (0.96, 1.36)	1.29 (0.98, 1.71)	0.50	1.19 (0.96, 1.47)	1.16 (0.95, 1.42)	0.74	1.27 (1.05, 1.52)	0.98 (0.75, 1.28)	0.09
6–7	0.96 (0.79, 1.16)	0.81 (0.59, 1.13)	0.15	0.83 (0.66, 1.04)	1.00 (0.79, 1.27)	0.11	0.94 (0.76, 1.16)	0.84 (0.63, 1.14)	0.53
7–9	Reference	Reference	Reference	Reference	Reference	Reference	Reference	Reference	Reference
$>$ 9	1.09 (0.82, 1.45)	1.55 (1.04, 2.30)	0.38	1.39 (1.00, 1.94)	1.08 (0.79, 1.48)	0.22	1.16 (0.88, 1.52)	1.15 (0.75, 1.78)	0.96
**The number of unrested days per week**									
$<$ 1	Reference	Reference	Reference	Reference	Reference	Reference	Reference	Reference	Reference
1–2	1.15 (0.93, 1.42)	1.18 (0.84, 1.66)	0.65	1.22 (0.95, 1.58)	1.13 (0.87, 1.46)	0.35	1.28 (1.02, 1.62)	1.07 (0.76, 1.50)	0.27
3–4	1.33 (1.08, 1.64)	1.13 (0.79, 1.62)	0.36	1.30 (0.97, 1.73)	1.27 (1.00, 1.60)	0.65	1.30 (1.03, 1.63)	1.24 (0.90, 1.72)	0.75
5–7	1.20 (0.98, 1.45)	1.72 (1.26, 2.33)	0.25	1.12 (0.87, 1.43)	1.49 (1.19, 1.85)	0.13	1.26 (1.03, 1.55)	1.55 (1.16, 2.07)	0.26

^a^ According to consistent solid fuels use and adjusted for age, sex, education, marital status, household expenditure, active or passive smoking, urbanicity and cooking location within household.

^b^ *P* value was calculated using the product term between the exposure and the modifying variable.

There were no apparent differences found in analyses stratified by sex (all interaction *p* values > 0.10). Only for the most severe group of 5–7 unrested days the effect for long-term solid fuels use appeared to be stronger on women than on men (Table 3). Overall, there was no apparent heterogeneity by rural or urban residence observed (Table 3). The estimated effect of consistent solid fuels use and insufficient sleep was stronger among rural residents (OR = 1.27 95% CI 1.05, 1.52; interaction *p* value = 0.09).

### Associations between consistent solid fuels use and co-occurrence of sleep problems and depression

Solid fuels usage in three surveys was associated with sleep problems among participants without depression (OR = 1.19 95% CI 1.03, 1.37). A two-fold higher odd was observed for the co-occurrence of both sleep problems and depression (OR = 2.11 95% CI 1.74, 2.55) among long-term solid fuels users compared with non-users (Table 4).

**Table 4:** Associations between solid fuels use for cooking and sleep problem with the co-occurance of depression.

Outcomes^a,b^	Cleaner fuels use (2861)	Consistent solid fuels use (reported in all surveys) (2491)	OR (95% CI) ^*c*^	*P* value for cumulative years of exposure^d^
Without sleep problems and depression	1406	923	Reference	Reference
Sleep problems only	1084	891	1.19 (1.03, 1.37)	0.06
Depression only	68	111	1.92 (1.34, 2.74)	< 0.01
With both sleep problems and depression	303	566	2.11 (1.74, 2.55)	< 0.01

^a^Participants defined as having sleep problems when they reported either having insufficient (≤ 6 hrs) or excessive (≥ 9 hrs) sleep durations or higher frequency of feeling unrested (≥ 3 days in a week).

^b^596 participants with missing data on depression were excluded in the statistical analyses.

^c^According to consistent solid fuels use and adjusted for age, sex, education, marital status, household expenditure, active or passive smoking, urbanicity and cooking location within household.

^d^*P* value was calculated using the cumulative years of solid fuels use exposure (0, 2, 4 and 6 years) fitted as a continuous variable.

### Sensitivity analyses

Among those who enrolled in 2011, 63.6% also completed the subsequent surveys in 2013 and 2015. Adults less than 65 years, married, living in rural area and solid fuel users at baseline were slightly more likely to have participated in all three surveys (Supplementary Table 1). Our findings remain unchanged using the IPSW-adjusted estimates suggesting that influence from selection bias is minimal (Supplementary Table 2). Our main results also did not markedly change when we excluded natural gas users in the reference group (Supplementary Table 3). The cross-sectional associations between solid fuels use and sleep health in each study wave is presented in Supplementary Table 4. Solid fuels use was associated with higher frequencies of unrested days of sleep in all three waves. For sleep duration, solid fuels use was also associated with excessive sleep in the wave 2011 and wave 2015 and with insufficient sleep in the wave 2011 and 2013 (Supplementary Table 4). Among solid fuel users identified in 2011, those who switched to cleaner fuels during the study period had lower odds for excessive sleep (average sleep hours > 9 h; OR = 0.76 95% CI 0.60, 0.97) in 2015. The point estimates for days feeling unrested were also lower for those who switched to cleaner fuels use but the 95% CIs were wide (Supplementary Table 5).

## Discussion

In this study, we analyzed a nationally representative database of adults over 45 years in China with a high proportion of households using solid fuels for cooking. Our findings suggest that a longer duration of using solid fuels for cooking was associated with self-reported poor sleep health as indicated by non-optimal duration of sleep and higher frequencies of unrested days. Some effect sizes observed appear to be stronger for the population 65 years and older, but confirmation of our findings with improved exposure measures and clinically validated sleep outcomes are warranted.

Previous epidemiological studies of air pollution and sleep problems have predominantly focused on outdoor air pollution^24,26,27,50^ and short-term responses^25^. Limited studies focused on the association between solid fuels use and sleep health. In children, two intervention studies conducted in Peru that replaced household biomass fuel with cleaner fuel kitchen stoves suggested improved symptoms of sleep apnea and respiratory problems^30,31^. In adults, the China Hainan Centenarian Cohort Study of 1,616 elderly aged 80 years and older showed that household air pollution from solid fuel combustion increased the risk of having poor sleep quality measured by the Pittsburgh sleep quality index (PSQI)^32^. Moreover, a cross-sectional study of 2,197 middle-aged adults recruited from employees working in a Machinery Company in Liuzhou, China, reported exposure of emissions from cooking oil combustion affected poor sleep quality measured by PSQI^33^. Our findings, which are based on a large sample drawn from representative data across multiple regions in China, add to the literature and suggest that solid fuels used for cooking might also contribute to impaired sleep health in middle-aged and elderly Chinese residents.

Studies have shown that solid fuels use could generate elevated indoor exposure to PM, NO_2_ and SO_2_^51,52^. A study in Yunnan Province, China, measured indoor NO_2_ in households from 30 villages found that 24-h averaged NO_2_ levels reached 132 ppb for households using smokeless coal and 111 ppb for households using smoky coal at home^53^. A study in India evaluating homes in 51 villages reported the 24-h average PM_2.5_ concentration of 372 µg/m^3^ in kitchens where solid fuels were used^54^. The concentrations of these pollutants were above World Health Organization (WHO) air quality guideline values, which recommend 25 µg/m^3^ for 24-h mean PM_2.5_ concentrations and 40 ppb for annual mean NO_2_ levels^55^.

The biological mechanism of how indoor air pollutants affect sleep health is still unclear, while some suggestive mechanisms have been proposed. Studies have suggested exposure to PM_2.5_ can affect neurobehavioral functions of central nervous systems which regulate sleep in human and animal models^22,56,57^. Long-term exposure to excess air pollutants could also damage intact mucociliary nasal epithelium and increase the risk of respiratory tract inflammation, leading to breathing problems and sleep-disorder^23^. Excess concentrations of O_3_, SO_2_, PM_10_ and PM_2.5_ exposure have been demonstrated to increase proinflammatory mediators including interleukin-6, and changes in the inflammatory cytokine levels may affect sleep quality^23,58,59^. Long-term PM_2.5_ exposure could also increase the stress level and anxiety or depressive symptoms^60,61^ and subsequently disrupt normal sleep. Considering the complexity of mixture air pollution, further studies are needed to elucidate the mechanisms of actions for air pollution impacts on sleep disorders.

Previous studies using CHARLS data have suggested shorter nighttime sleep duration < 6 h was associated with a high risk of depressive symptoms in Chinese elderly^62^, and a higher proportion of current household solid fuels users reported depression symptoms measured by CES-D scale compared with clean fuels users^46^. The relationship between sleep problems and depression is likely bi-directional^63^. Our analyses that considered both sleep and depressive symptoms suggested a stronger association for co-occurrence of sleep problems and depression among long-term solid fuels use, while a weaker association was also noted for sleep problems only without depression. Future research of indoor and outdoor air pollution should explore the role of sleep problems as a potential mediating factor that leads to depression or other major chronic diseases in older adults^4,64^.

Household solid fuels use is the primary pollutant source of indoor air pollution in developing countries^34^. In China, about 170 million urban residents and 490 million rural residents used solid fuels for cooking according to 2010 census data^65^. Energy issues have drawn more and more attention. The government and residents have taken measures to promote cleaner fuels like electric, natural gas, liquefied petroleum gas for cooking^66^. Repeated surveys in CHARLS from 2011 to 2015 also suggested the use of cleaner fuel types has become more common among older residents in China. However, a high cost associated with cleaner fuels use may be a barrier for the low-income households to adopt the change in cooking fuels. Continuing efforts in reducing solid fuels use for low-income rural residents in China are needed.

Our studies have several strengths. First, we analyzed data from a large study with three repeated surveys from a representative sampling of 150 counties/districts in China, including main Chinese provinces and cities. The prevalence of solid fuels use for cooking is high in this population. To our knowledge, there was no similar study focusing on the association between solid fuels use and sleep health in such a large population in China. The repeated surveys also allowed us to evaluate the duration of use and the change from solid to cleaner fuels as the primary household cooking fuels. A range of potential confounding factors were considered in analyses. The large sample size also allowed us to evaluate potential effect modification by age, sex and urbanicity, and study the co-occurrence of sleep problems and depression.

Our study has several limitations. First, we did not have any direct measurements of indoor air pollutant levels from cooking fuels. However, this would require repeated measurements to capture long-term exposure effects, which is less feasible for a large population study. Our analyses primarily addressed cumulative exposure utilizing data from three waves of CHARLS. The cross-sectional association between solid fuels usage and sleep outcomes at each wave might reflect short-term exposure effect, but the results can be sensitive to reverse causality bias. For example, sleep problems can affect the participants’ lifestyles and behaviors. Second, the data we used was primary cooking fuels without considering heating fuels as another exposure source. Solid use for heating is an important source of indoor air pollution, especially for northern China during the winter season. Some household factors, including the kitchen design and ventilation, might modify indoor pollution level from cooking^67^. The CHARLS database also does not provide outdoor air pollution measures for analyses. Sleep outcomes were self-reported and there was no availability of clinical measures or information regarding sleep medication use. More in-depth assessments of sleep quality, such as the use of polysomnography or objective sleep-scale measures, coupled with novel wearable tools to characterize personal environmental exposure profiles^68^, could be employed to advance our understanding of the relationship between indoor air pollution and sleep health. Finally, the influence from uncontrolled confounding cannot be ruled out. Some uncontolled variables, such as diets, physical activities, and other social and physical environmental factors, could affect sleep health. These factors, however, might not strongly correlated with the choice of fuels type for cooking after controlling socio-economical differences in the statistical analyses.

## Conclusions

In this study based on the three waves of CHARLS from 2011–2015, we found consistent solid fuels use for cooking during the study period was associated with self-reported poor sleep health in Chinese adults 45 years and older. Strategies to promote cleaner fuels for cooking are needed for Chinese elderly residents. Future studies are recommended to investigate the impact of specific fuel types and the biological mechanism linking air pollutants released from solid fuels to sleep disorders etiology.

## Supplementary Information

Supplementary Information.

## Data Availability

The datasets used and analyzed during the current study are available and assessable from the China Health and Retirement Longitudinal Study (CHARLS, http://charls.pku.edu.cn/zh-CN).
