# Supplementary material for: Solid fuels use for cooking and sleep health in adults aged 45 years and older in China
Source: Sci Rep. 2021 Jun 25;11:13304. doi: 10.1038/s41598-021-92452-0 (PMC8233310; doi:10.1038/s41598-021-92452-0)
Supplement: Supplementary file 1 — Supplementary Information. [file 41598_2021_92452_MOESM1_ESM.docx]

**Solid fuels use for cooking and sleep health in adults aged 45 years and older in China**

Haiqing Yu^1^, Jiajun Luo^1^, Kai Chen^1^, Krystal J. Godri Pollitt^1,2^, and Zeyan Liew^1,2,*^

^1^ Department of Environmental Health Sciences, Yale School of Public Health, New Haven, CT, 06510, USA.

^2^ Yale Center for Perinatal, Pediatric, and Environmental Epidemiology, Yale School of Public Health, New Haven, CT, 06510, USA.

**Correspondence:** Address correspondence to Zeyan Liew, Department of Environmental Health Sciences, Yale School of Public Health. Full address: 60 College Street, New Haven CT 06510. Tel: 203-764-9727. Email: [zeyan.liew@yale.edu](mailto:zeyan.liew@yale.edu)

**Supplementary Table 1.** The distribution of baseline study characteristics among participants who answered all three surveys from 2011 to 2015 and those with missing at least one follow-up surveys

| Variables | Participants completed all three waves (N=10511), N (%) | Participants completed 2011 but were missing in later waves  (N=6022), N (%) |
| --- | --- | --- |
| Fuels use for cooking |  |  |
| Solid fuels | 5739 (54.6) | 3117 (51.8) |
| Cleaner fuels | 4772 (45.4) | 2905 (48.2) |
| Average hours of sleep at night |  |  |
| $<$6 | 2805 (28.4) | 1601 (29.8) |
| 6-7 | 2144 (21.7) | 1113 (20.7) |
| 7-9 | 4131 (41.9) | 2173 (40.4) |
| $>$9 | 789 (8.0) | 485 (9.0) |
| Missing | 642 | 650 |
| The number of unrested days per week |  |  |
| <1 | 4875 (49.6) | 2584 (48.4) |
| 1-2 | 1679 (17.1) | 833 (16.5) |
| 3-4 | 1415 (14.4) | 812 (15.2) |
| 5-7 | 1859 (18.9) | 1065 (19.9) |
| Missing | 683 | 678 |
| Age (years) |  |  |
| 45-65 | 8155 (79.3) | 3963 (67.5) |
| Over 65 | 2133 (20.7) | 1911 (32.5) |
| Missing | 223 | 148 |
| Sex |  |  |
| Male | 5165 (49.2) | 2752 (45.8) |
| Female | 5341 (50.8) | 3263 (54.3) |
| Missing | 4 | 7 |
| Urbanicity of the living place |  |  |
| Rural | 6543 (62.3) | 3293 (54.7) |
| Urban | 3968 (37.8) | 2729 (45.3) |
| Expenditure in 2011 (RMB) |  |  |
| ≤10,000 | 7358 (74.9) | 4056 (73.6) |
| 10,000-50,000 | 2231 (22.7) | 1304 (23.7) |
| 50,000-100,000 | 151 (1.5) | 92 (1.7) |
| $>$100,000 | 79 (0.8) | 62 (1.1) |
| Missing | 692 | 508 |
| Educational level |  |  |
| No formal | 4447 (42.4) | 2936 (48.9) |
| ≤12 years | 5836 (55.6) | 2874 (47.8) |
| ≥12 years | 218 (2.1) | 200 (3.3) |
| Missing | 10 | 12 |
| Marital status |  |  |
| Married | 8767 (83.4) | 4524 (75.1) |
| Not married | 1744 (16.6) | 1498 (24.9) |
| Smoking |  |  |
| Active | 3923 (38.4) | 1986 (34.7) |
| Passive | 2911 (28.5) | 1506 (26.3) |
| Never | 3388 (33.1) | 2239 (39.1) |
| Missing | 88 | 80 |
| Cooking location within household |  |  |
| Yes | 9624 (91.8) | 5398 (89.9) |
| No | 861 (8.2) | 608 (10.1) |
| Missing | 20 | 22 |

**Supplementary Table 2.** Associations between solid fuels use for cooking and sleep health, adjustment for selection using the inverse probability selection weight (IPSW)

| Outcomes | Inconsistent solid fuels use (reported in 1 or 2 surveys)  vs. cleaner fuels use | Consistent solid fuels use (reported in all surveys) vs. cleaner fuels use | P-value for cumulative years of exposure *^b^* |
| --- | --- | --- | --- |
|  | OR (95% CI) *^a^* | OR (95% CI) *^a^* |  |
| Average hours of sleep at night |  |  |  |
| $<$6 | 1.01 (0.85, 1.20) | 1.14 (0.95, 1.37) | 0.27 |
| 6-7 | 0.96 (0.79, 1.15) | 0.90 (0.73, 1.10) | 0.24 |
| 7-9 | Reference | Reference | Reference |
| $>$9 | 1.03 (0.78, 1.35) | 1.25 (0.94, 1.67) | 0.10 |
| The number of unrested days per week |  |  |  |
| $<$1 | Reference | Reference | Reference |
| 1-2 | 1.12 (0.91, 1.39) | 1.17 (0.93, 1.46) | 0.15 |
| 3-4 | 1.08 (0.87, 1.33) | 1.28 (1.02, 1.60) | 0.03 |
| 5-7 | 1.20 (1.00, 1.45) | 1.27 (1.04, 1.55) | 0.11 |

^a^ Adjusted for age, sex, education, marital status, household expenditure, active or passive smoking, urbanicity, and cooking location within household. Models included the IPSW procedure to account for the predicted probability of participation in all three surveys.

^b^ P-value was calculated using the cumulative years of solid fuels use exposure (0, 2, 4, and 6 years) fitted as a continuous variable.

**Supplementary Table 3.** Associations between solid fuels use and sleep health, excluding natural gas use in reference

| Outcomes | Inconsistent solid fuels use (reported in 1 or 2 surveys)  vs. cleaner fuels use excluding natural gas | Consistent solid fuels use (reported in all surveys) vs. cleaner fuels use excluding natural gas | P-value for cumulative years of exposure *^b^* |
| --- | --- | --- | --- |
|  | OR (95% CI) *^a^* | OR (95% CI) *^a^* |  |
| Average hours of sleep at night |  |  |  |
| $<$6 | 0.97 (0.81, 1.15) | 1.12 (0.94,1.33) | 0.37 |
| 6-7 | 0.88 (0.73, 1.06) | 0.86 (0.71 1.04) | 0.10 |
| 7-9 | Reference | Reference | Reference |
| $>$9 | 0.93 (0.71, 1.21) | 1.11 (0.85, 1.44) | 0.48 |
| The number of unrested days per week |  |  |  |
| <1 | Reference | Reference | Reference |
| 1-2 | 1.22 (0.98, 1.51) | 1.25 (1.01, 1.56) | 0.02 |
| 3-4 | 1.07 (0.87, 1.32) | 1.29 (1.04, 1.59) | 0.01 |
| 5-7 | 1.27 (1.04, 1.54) | 1.38 (1.13, 1.68) | 0.02 |

^a^ According to solid fuels use and adjusted for age, sex, education, marital status, household expenditure, active or passive smoking, urbanicity, and cooking location within household.

^b^ P-value was calculated using the cumulative years of solid fuels use exposure (0, 2, 4 and 6 years) fitted as a continuous variable

**Supplementary Table 4.** Cross-sectional associations between solid fuels use for cooking and sleep health, by study waves

| Outcomes | Solid fuels use vs. clean fuels use in 2011  (N=8668) | Solid fuels use vs. clean fuels use in 2013  (N=8668) | Solid fuels use vs. clean fuels use in 2015  (N=8668) |
| --- | --- | --- | --- |
|  | OR (95% CI) *^a^* | OR (95% CI) *^a^* | OR (95% CI) *^a^* |
| Average hours of sleep at night |  |  |  |
| $<$6 | 1.18 (1.05, 1.34) | 1.19 (1.04, 1.36) | 1.10 (0.99, 1.24) |
| 6-7 | 1.02 (0.90, 1.17) | 0.93 (0.80, 1.08) | 0.94 (0.82, 1.07) |
| 7-9 | Reference | Reference | Reference |
| $>$9 | 1.28 (1.05, 1.56) | 1.11 (0.88, 1.41) | 1.22 (1.02, 1.45) |
| The number of unrested days per week |  |  |  |
| <1 | Reference | Reference | Reference |
| 1-2 | 1.15 (0.99, 1.32) | 1.10 (0.94, 1.28) | 1.14 (0.99, 1.31) |
| 3-4 | 1.06 (0.91, 1.23) | 1.38 (1.17, 1.64) | 1.15 (1.00, 1.32) |
| 5-7 | 1.26 (1.10, 1.45) | 1.26 (1.08, 1.47) | 1.12 (1.00, 1.25) |

^a^ Adjusted for age, sex, education, marital status, household expenditure, active or passive smoking, urbanicity and cooking location within household in each wave.

**Supplementary Table 5.** Associations between a change from solid to cleaner fuels use for cooking and sleep health

| Outcomes | Solid fuel users in 2011 who switched to cleaner fuels use in later waves (N=1604) compared with consistent solid fuel users during 2011-2015 (N=2671) | Solid fuel users in 2011-2013 who switched to cleaner fuels use in the 2015 wave (N=743) compared with consistent solid fuel users during 2011-2015 (N=2671) |
| --- | --- | --- |
|  | OR (95% CI) *^a^* | OR (95% CI) *^a^* |
| Average hours of sleep at night |  |  |
| $<$6 | 0.90 (0.77, 1.05) | 0.83 (0.68, 1.01) |
| 6-7 | 1.08 (0.90, 1.29) | 0.94 (0.74, 1.19) |
| 7-9 | Reference | Reference |
| $>$9 | 0.76 (0.60, 0.97) | 0.68 (0.49, 0.95) |
| The number of days unrested per week |  |  |
| $<$1 | Reference | Reference |
| 1-2 | 0.92 (0.76, 1.12) | 0.96 (0.75, 1.24) |
| 3-4 | 0.87 (0.71, 1.05) | 0.95 (0.74, 1.22) |
| 5-7 | 0.95 (0.80, 1.13) | 0.82 (0.65, 1.03) |

^a^ Adjusted for age, sex, education, marital status, household expenditure, active or passive smoking, urbanicity, and cooking location within household.
